# Dispositional Mindfulness and Attentional Control: The Specific Association Between the Mindfulness Facets of Non-judgment and Describing With Flexibility of Early Operating Orienting in Conflict Detection

**DOI:** 10.3389/fpsyg.2018.02359

**Published:** 2018-11-29

**Authors:** Lin Sørensen, Berge Osnes, Endre Visted, Julie Lillebostad Svendsen, Steinunn Adolfsdottir, Per-Einar Binder, Elisabeth Schanche

**Affiliations:** ^1^Department of Biological and Medical Psychology, University of Bergen, Bergen, Norway; ^2^KGJebsen Center for Neuropsychiatric Disorders, Bergen, Norway; ^3^Bjørgvin District Psychiatric Centre, Haukeland University Hospital, Bergen, Norway; ^4^Department of Clinical Psychology, University of Bergen, Bergen, Norway; ^5^Division of Psychiatry, Haukeland University Hospital, Bergen, Norway; ^6^Department of Child and Adolescent Psychiatry, Haukeland University Hospital, Bergen, Norway

**Keywords:** mindfulness, five facet mindfulness questionnaire (FFMQ), attention networks, attention network test (ANT), revised attention network test

## Abstract

**Background:** A state of mindfulness refers to a present-centered attentional awareness without judging. Being mindful seems to increase the ability to be flexible and adaptive in attention focus according to situational contingencies. The way mindfulness affects such attentional control is often measured with three different but interacting attentional networks of alerting (preparedness), orienting (selection of stimulus), and conflict detection (suppression of irrelevant stimuli). In the current study, the aim was to study the effects of dispositional mindfulness on these attention networks, and specifically the effects on the interactions between these attention networks.

**Methods:** Fifty participants between 19 and 29 years old filled out the questionnaire Five Facet Mindfulness Questionnaire (FFMQ) and performed the revised version of the Attention Network Test (ANT-R). The five FFMQ facets of Describing, Non-Judgment, Orienting, Non-Reactivity, and Acting with Awareness were included as predictors in multiple linear regression analyses with the ANT-R scores of alerting, orienting, conflict detection, and the interaction scores of alerting by conflict detection and orienting by conflict detection as outcome variables, respectively.

**Results:** Higher dispositional mindfulness as measured with the five FFMQ facets predicted interaction scores (faster reaction times) of orienting by conflict detection, but none of the other ANT-R scores. It was specifically the FFMQ facets of Describing and non-judgment that predicted this lower interaction score of orienting by conflict detection.

**Conclusion:** Our findings indicate that being mindful is associated with a more flexible and efficient orienting attention. It is associated with a higher ability to disengage from salient stimuli that is irrelevant to pursue goal-directed behavior (conflict detection).

## Introduction

Mindfulness refers to a mental state characterized by a present-centered attentional awareness without judging (Bishop et al., 2004). A state of mindfulness affects the way we experience the context that we live in and how we relate to these experiences, which seem to protect against and relieve both mental and somatic health issues (Keng et al., 2011; De Vibe et al., 2017). A central feature of mindfulness is the flexible and adaptive self-regulation of attention in accordance with contextual contingencies (Bishop et al., 2004; Malinowski, 2013; Tang et al., 2015). This comprises the ability to allocate attentional resources and handle conflicts between action options. Such an ability to voluntarily suppress some responses in favor of others is important in goal-directed behavior (attentional control; Posner and Petersen, 1990; Miller and Cohen, 2001; Cooper, 2010). Improved attentional control as an effect of mindfulness is suggested to help people act with more autonomy in pursuing intrinsic goals and increase the experience of overall well-being (Lutz et al., 2008; Schultz and Ryan, 2015).

The predominant focus in neuroscience has been on the effects of intentional mindfulness training on attentional control, and less on individual differences in dispositional mindfulness (Tang et al., 2016; Wheeler et al., 2016). Pre-existing tendencies to be mindful are shown to associate with better psychological self-regulation (Brown and Ryan, 2003), and may as such influence who is showing an improved effect of intentional mindfulness training (Tang et al., 2016; Wheeler et al., 2016). It is therefore interesting to study if dispositional mindfulness relates to attentional control in similar ways as effects of intentional mindfulness.

Dispositional mindfulness is typically measured with self-report questionnaires such as the multi-faceted “Five Facet Mindfulness Questionnaire” (FFMQ; Baer et al., 2006) or the “Mindfulness Attention Awareness Scale” (MAAS) (Brown and Ryan, 2003). The FFMQ is reckoned to give a broader assessment of mindfulness, in both assessing the attentional and attitudinal components (facets) of the construct, whereas the MAAS is predominantly focused on the attentional component (Brown and Ryan, 2003; Zhuang et al., 2017). The FFMQ includes five facets of mindfulness: Observing (the observation of inner experiences, reactions, and sensations); Non-Judging (observing inner experiences without judging); Describing (putting into words observations of inner experiences of perceptions, thoughts, feelings, sensations, and emotions); Non-Reactivity (not reacting to inner experiences); and Acting with Awareness (the ability to voluntarily focus attention).

Effects of mindfulness have often been studied by using the Attention Network Theory that distinguishes between three interacting networks of alerting, orienting and conflict detection (Chiesa et al., 2011; Tang et al., 2015; see also Posner and Petersen, 1990; Petersen and Posner, 2012): *Alerting* refers to the preparedness for mentally effortful monitoring, detection, and reaction to action conflicts, either tonic due to requirements of vigilance or physically as a response to warning signals. *Orienting* refers to the selection of valid stimuli/action options in the presence of numerous sensory stimuli. *Conflict detection* refers to the monitoring and suppression of salient but non-relevant stimuli in favor of task-relevant stimuli, often referred to as executive control (Posner, 2008; Fan et al., 2009; Petersen and Posner, 2012; Mackie et al., 2013). Attentional control comprises the interactions between the early operating discrimination of valid/invalid stimulus and readiness and the later operating process of the conflict detection. Improved conflict detection as an effect of mindfulness training has been observed in several studies (Wenk-Sormaz, 2005; Chan and Woollacott, 2007; Slagter et al., 2007; Tang et al., 2007; Moore and Malinowski, 2009; Ainsworth et al., 2013). It has also been observed a change toward a more efficient early attention allocation of orienting following mindfulness training (Jha et al., 2007), whereas positive effects of such training on alerting have predominantly been found in long-term meditators (Jha et al., 2007; Pagnoni and Cekic, 2007; MacLean et al., 2010; Tang et al., 2012).

The relationship between dispositional mindfulness and attentional control shows inconsistent results across the few studies that have been conducted (Josefsson and Broberg, 2011; Ainsworth et al., 2013; Di Francesco et al., 2017; Jaiswal et al., 2018). These inconsistencies seem to stem from applying different versions of the Attention Network Test (ANT), which measures the three attention networks, and different approaches in measuring dispositional mindfulness. For instance, dispositional mindfulness, as measured by dividing into high and low mindfulness according to the mean total score of the MAAS, was found not to differentiate performance on the original ANT (Fan et al., 2002) or on other attentional control tasks (a Stroop color-word interference task and a working memory task) (Jaiswal et al., 2018). Another study (Ainsworth et al., 2013) using the MAAS, found dispositional mindfulness to be related to more efficient conflict detection on an emotional ANT. Using the FFMQ, a recent study (Di Francesco et al., 2017) found a relationship between dispositional mindfulness and attentional control measured with a modified version of the original ANT with auditory warning signals. However, the latter study found opposite of what is expected that higher scores on the facets of Observing and Acting with Awareness associated with slower reaction times. In contrast a study (Josefsson and Broberg, 2011) not using the ANT, found that the FFMQ facet of Describing associated with higher accuracy on a sustained attention task and a more efficient conflict detection on a Stroop color-word interference task both among experienced meditators and non-meditators.

Effects of dispositional and intentional (training) mindfulness have to date predominantly focused on the three attention networks as single scores and main effects. However, there are some indications of possible interaction effects between the early operating attention selection and the conflict detection (Malinowski, 2013). For instance, an event-related potential (ERP) study (Moore et al., 2012) showed that effects of mindfulness training seemed to relate to increased flexibility of the early attention allocation, specifically perceptual discrimination (orienting) (an increased N2 component amplitude and reduced P3 component amplitude; Moore et al., 2012), and not conflict detection *per se*. These neuronal effects of early processing perceptual discrimination were observed primarily for the incongruent conflict detection on a Stroop color-word interference task (see Malinowski, 2013). Another, recent study (Zhuang et al., 2017) investigated the correlations between individual differences in dispositional mindfulness with the FFMQ facets and neuroanatomical structures. They found that the FFMQ facet of Describing correlated with the brain volume size of dorsolateral prefrontal cortex, the right inferior parietal lobule, and the left superior frontal cortex among others involved in self-awareness, emotion regulation (cognitive reappraisal), and attentional control (see Zhuang et al., 2017). Interestingly though, two of the brain structures (the dorsolateral prefrontal cortex and the right inferior parietal lobule) correlating with the Describing facet are part of the fronto-parietal network (Corbetta and Shulman, 2002; Raz and Buhle, 2006; Dosenbach et al., 2007; Posner and Rothbart, 2007; Singh-Curry and Husain, 2009; Tang et al., 2015) involved in the interaction between orienting and conflict detection (Petersen and Posner, 2012) Further, also, the non-judgment facet of the FFMQ correlated with the surface area of the superior prefrontal cortex involved in self-awareness and attention.

In the current study, we investigated the relationship between the five facets of the FFMQ and attentional control as measured with the revised Attention Network Test (ANT-R; Fan et al., 2009; Mackie et al., 2013) in a sample previously naïve to meditation. The ANT-R presents a flanker conflict condition either on the right or left hand sides of the screen, whereas the original ANT only presented this condition in the center of the screen. The revision allows for studying the effect of spatial cuing, indicating valid or invalid spatial appearance of the flanker stimuli (see Posner, 1980), on the conflict detection. Thus, in addition to the attention network scores of orienting and conflict detection, as generated with the original ANT, an interaction score is generated of orienting by conflict detection (Fan et al., 2009; Mackie et al., 2013). The orienting score is calculated with the reaction times of detecting flanker conflicts on trials with preceding valid spatial cues minus trials with preceding invalid spatial cues. The interaction score of orienting by conflict detection, in contrast to the single orienting score, measures specifically if the effect of such a spatial cue (valid/invalid) appeared on a congruent conflict condition (e.g., flanker arrows pointing in the same direction as the target arrow) or on an incongruent conflict condition (e.g., flanker arrows pointing in an opposite direction of the target arrow). The inclusion of a task that allows for studying behavioral interactions between early attention allocation and conflict detection made it possible to test if higher levels of dispositional mindfulness would relate to a lower (more efficient) interaction score between the early operating orienting and the conflict detection. Building on the studies of Moore et al. (2012) and Zhuang et al. (2017), we expected that the FFMQ facets of Describing and Non-Judgment to explain this association (see Zhuang et al., 2017). Since Acting with Awareness is the facet that seems to specifically tap into self-report of attentional control, we also expected this to relate with the orienting by conflict detection interaction score.

## Materials and Methods

### Participants

The participants were recruited through internal announcements by email and on a university webpage at the University of Bergen, Norway. The study aimed to investigate the association between mindfulness and attention networks and psychophysiological measures considered to be included in a larger planned randomized controlled study (see also Sørensen et al., 2015; Svendsen et al., 2016; Visted et al., 2017). Exclusion criteria included presence of a severe psychiatric illness, cardiac illness, use of sedative or psychoactive medication and previous experience with mindfulness, i.e., attended mindfulness courses, participated in mindfulness retreats or received other kinds of formalized mindfulness training. Fifty-three students were recruited. Fifty of them performed the ANT-R in the age range of 19–29 years of age (mean age = 23.48; *SD* = 2.21; 74% females). The participants gave informed consent in accordance with the Helsinki declaration and by the Regional Ethics Committee (South East: Study number 2014/148).

### Procedure

The participants were asked to refrain from intake of nicotine and caffeine 3 h prior to performing the ANT-R. They were also asked to refrain from alcohol or psychoactive drugs the day of the experiment. They performed the ANT-R approximately at the same time in the afternoon (both pre- and post-mindfulness training), and filled out the FFMQ together with other questionnaires in the same session.

### The FFMQ

The FFMQ (Baer et al., 2006) consist of 39 items measuring mindfulness. The items are rated on a five-point Likert scale from 1 (“never or very rarely true”) to 5 (“very often or always true”), measuring five factors (facets): Observing (e.g., “I notice the smells and aromas of things”), Describing (e.g., “I am good at finding words to describe my feelings”), Acting with Awareness (e.g., “I find myself doing things without paying attention”; reverse scored item), Non-Judgment of inner experience (e.g., “I think some of my emotions are bad or inappropriate and I should not feel them”; reverse scored item), and Non-Reactivity to inner experience (e.g., “I perceive my feelings and emotions without having to react to them”).

The FFMQ and its facets have shown good construct validity in distinguishing the scores from other, associated phenomena (Baer et al., 2006, 2008). The five facets shows a high internal consistency, with alpha coefficients ranging from 0.76 to 0.91 (Baer et al., 2006). The test-retest reliability has also been demonstrated to be good to excellent in a Dutch sample (Veehof et al., 2011). In the current study, we used a Norwegian translation of the FFMQ (Dundas et al., 2013). The range in each facet score was: Observe (1.63–4.75; mean = 3.21; *SD* = 0.71); Describing (1.00–5.00; mean = 3.46; *SD* = 0.94); Non-Judgment (1.25–5.00; mean = 2.89; *SD* = 0.84); Non-Reactivity (1.29–4.71; mean = 2.54; *SD* = 0.70); and Acting with Awareness (1.13–4.50; mean = 2.58; *SD* = 0.75). The internal consistency (alpha coefficients) of the five facets was calculated in the current study and found to be high: Observe = 0.75; Describing = 0.94; Non-Judgment = 0.89; Non-Reactivity = 0.87; and Acting with Awareness = 0.87.

### The Revised Attention Network Test (ANT-R)

The ANT-R (Fan et al., 2009; Mackie et al., 2013) is a revised version of the attention network test (Fan et al., 2002), in which a flanker condition is combined with varying cue conditions. The revised version includes two target conditions (congruent and incongruent) and three cue conditions (no cue, double cue, spatial cue). In the current study, the reaction time generated scores for the attention networks of alerting, orienting, and conflict detection, in addition to the two interaction scores of alerting by conflict detection and orienting by conflict detection, were included (see Table 1).

**Table 1:** Descriptive information about the ANT-R scores.

Attention networks	Variable score	Measures	Operational score calculation	*M* (*SD*)
Alerting	Alerting	Tonic^a^ and phasic arousal (temporal cues)	RT_no cue – RT_double cue	42.66 (30.19)
Orienting	Validity effect	Endogenous^a^ and exogenous attention engagement (spatial cues)	RT_invalid cue – RT_valid cue	106.13 (37.44)
Cognitive control	Flanker conflict	Conflict processing (congruent and incongruent conditions)	RT fl. incongr. – RT fl. congr.	152.78 (48.17)
**Interaction scores**				
Alerting by flanker	Alert ing^∗^flanker	The effect of alerting (temporal) cues on the conflict processing^a^	(RT_no cue, fl. incongr. – RT_no cue, fl. congr.) – (RT_double cue, fl. incongr. – RT_double cue, fl. congr.)	17.34 (54.43)
Orienting by flanker	Validity^∗^flanker	The effect of orienting (spatial) cues on the conflict processing^a^	(RT_invalid cue, fl. incongr. – RT_invalid cue, fl. congr.) – (RT_valid cue, fl. incongr. – RT_valid cue, fl. congr.)	69.14 (47.48)

*RT, reaction time; fl., flanker; incongr., incongruent; congr., congruent. ^a^Lower scores indicate the intrinsic, self-regulated effect on alertness and orienting.*

Performing the ANT-R, the participants are presented with a target stimulus, a center arrow flanked by two arrows on each side. They are instructed to indicate the direction of the center arrow by pressing a key with the index finger for the left direction and with the ring finger for the right direction on a mouse. A congruent flanker condition is presented when the center arrow points in the same direction as the flanker arrows. An incongruent flanker condition is when there is a conflict of the center arrow pointing in another direction from the flanker arrows. A fixation cross is presented on all trials. This is followed by the appearance of the flanker condition on either the left hand side or on the right hand side of the screen for 500 ms. Different cue conditions are presented for 100 ms before the flanker condition appears, shown with boxes flashing. These cues could indicate the spatial presentation of the flanker condition (valid) or not (invalid), or alternatively, temporal cues can be presented by both the boxes on the right hand and left hand sides flashing (double cue), or no boxes would be flashing before the presentation of the flanker condition (no cue). The inter-stimulus-interval varies between the trials (0, 400, and 800 ms). The participants performed a practice task with step-by-step instructions for the cue and target conditions, and thereafter on 32 practice trials with the same timing limits as the real ANT-R. The participants in the current study performed the ANT-R with ≥ 80% accuracy.

The ANT-R comprises 4 runs of 72 test trials in each (see Fan et al., 2009 for more details). The task was run on a desktop PC, using E-Prime^TM^ software (Psychology Software Tools, Pittsburgh, PA, United States).

### Statistical Analyses

All the statistical analyses were performed with the SPSS, Version 24. Preliminary analyses were conducted to explore the role of age and gender on the scores of the FFMQ facets and the ANT-R. The relationship between the FFMQ facets and the ANT-R scores were explored. To test our hypothesis that dispositional mindfulness would predict a more efficient early operating orienting in conflict detection, we conducted multiple linear regression analyses to investigate the prediction of the FFMQ facets on the respective ANT-R scores of alerting, orienting, flanker conflict detection, alerting by flanker, and orienting by flanker^1^. The significance level was Bonferroni corrected according to conducting five main linear regression analyses with each of the ANT-R scores as outcome variables (0.05/5 = 0.01). Further, Posner (2008) has noted that the attention network scores can be complex to interpret across different samples. We therefore explored the relationship between each of the ANT-R scores and the trial raw scores of the cue validity x flanker conflict conditions (cue invalid x flanker congruent/incongruent conflict conditions and cue valid × flanker congruent/incongruent conflict conditions) (see Supplementary Table 1).

## Results

### Preliminary Analyses

Age did not correlate with the FFMQ facets (Observing, Describing, Non-Judgment, Non-Reactivity, or Acting with Awareness) or with the ANT-R scores (alerting, orienting, conflict detection, alerting by conflict detection or orienting by conflict detection). Further, independent samples *t*-tests showed that females reported higher scores on the FFMQ Non-Reactivity facet [*t*(48) = −2.20; *p* = 0.03]. No gender differences appeared on the other FFMQ facets or the ANT-R scores.

All of the FFMQ facets correlated with each other, except for that both the facets of Observing and Describing did not correlate with the Non-Judgment facet (see Table 2). Further, among the ANT-R scores, alerting correlated with the interaction of alerting by conflict detection and orienting correlated with the interaction of orienting by conflict detection. Alerting and orienting did not correlate with conflict detection, nor did conflict detection correlate with the interactions of alerting by conflict detection or orienting by conflict detection.

**Table 2:** Bivariate correlations between the FFMQ facets and the ANT-R scores.

			2	3	4	5	6	7	8	9	10	Age
FFMQ	1	Non-judging	0.23	0.20	0.51^∗∗^	0.51^∗∗^	−0.07	−0.12	0.09	0.22	−0.25	−0.11
	2	Describing		0.38^∗∗^	0.34^∗^	0.40^∗∗^	0.17	−0.24	−0.02	>0.01	−0.35^∗^	0.15
	3	Observing			0.53^∗∗^	0.50^∗∗^	−0.07	0.01	0.17	−0.12	0.13	0.17
	4	Acting with awareness				0.50^∗∗^	−0.07	0.06	0.04	0.07	0.10	−0.12
	5	Non-reactivity					0.01	0.02	−0.03	−0.03	0.02	−0.03
ANT-R	6	Alerting						0.06	0.19	0.29^∗^	−0.13	−0.23
	7	Orienting							−0.15	−0.01	0.61^∗∗^	0.10
	8	Conflict detection								−0.03	−0.07	−0.21
	9	Alerting by conflict detection									0.13	−0.15
	10	Orienting by conflict detection										−0.02

*N = 50; ^∗∗^p < 0.01; ^∗^p < 0.05.*

Only the FFMQ facet of Describing correlated with the ANT-R interaction score of orienting by conflict detection (see Table 2). Otherwise, none other of the FFMQ facets or ANT-R scores correlated with each other.

### The Prediction of the FFMQ Facets on the ANT-R Scores

In the regression analyses, all the five FFMQ facets were entered as predictors with the ANT-R scores of alerting, orienting, conflict detection, alerting by conflict detection, and orienting by conflict detection as outcome variables, respectively. The FFMQ facets significantly predicted the outcome of the ANT-R interaction score of orienting by conflict detection, and not the ANT-R scores of alerting, orienting, conflict detection, or alerting by conflict detection (see Table 3). The FFMQ facets of Describing and Non-Judgment specifically predicted a lower interaction score of orienting by conflict detection. Studying the relationship between the ANT-R scores and the raw scores of trials with spatial cues (see Supplementary Table 1) showed that the interaction score of orienting by flanker correlated positively with faster reaction times on trials presenting a flanker incongruent conflict preceded by invalid spatial cues. This indicates that an ability to be non-judgmental and/or to describe sensations and experiences associated with a more efficient (faster reaction times) ability to disengage from invalid cues to re-direct attention and detect incongruent flanker conflicts.

**Table 3:** The prediction of the FFMQ facets on the ANT-R outcome scores.

Model	Outcome score	Predictors	*R*^2^	*F*	*p*	β	*t*	*p*
1	Alerting	Non-judging	0.06	0.59	0.71	−0.10	−0.52	0.61
		Describing				0.24	1.48	0.15
		Observing				−0.13	−0.70	0.49
		Acting with awareness				−0.07	−0.33	0.74
		Non-reactivity				0.06	0.31	0.76
2	Orienting	Non-judging	0.12	1.18	0.34	−0.24	−0.133	0.19
		Describing				−0.31	−1.97	0.06
		Observing				−0.02	−0.13	0.90
		Acting with awareness				0.21	1.11	0.28
		Non-reactivity				0.17	0.88	0.39
3	Conflict detection	Non-judging	0.07	0.63	0.68	0.2	1.06	0.30
		Describing				−0.01	−0.08	0.94
		Observing				0.29	1.54	0.13
		Acting with Awareness				−0.11	−0.54	0.59
		Non-reactivity				−0.21	−1.07	0.29
4	Alerting by conflict detection	Non-judging	0.09	0.86	0.51	0.29	1.57	0.13
		Describing				0.03	0.17	0.86
		Observing				−0.15	−0.79	0.43
		Acting with awareness				0.06	0.33	0.75
		Non-reactivity				−0.14	−0.74	0.46
5	Orienting by conflict detection	Non-judging	0.33	4.39	0.002^∗∗^	−0.44	−2.8	0.008^∗∗^
		Describing				−0.49	−3.54	0.001^∗∗^
		Observing				0.12	0.74	0.46
		Acting with awareness				0.32	1.9	0.06
		Non-reactivity				0.22	1.34	0.19

*^∗∗^Bonferroni corrected alpha level of p < 0.01.*

## Discussion

In the current study we aimed to investigate the relationship between dispositional mindfulness and attentional control. It is previously shown that mindfulness training increases the efficiency of the attention networks of orienting (Jha et al., 2007) and conflict detection (Wenk-Sormaz, 2005; Chan and Woollacott, 2007; Slagter et al., 2007; Tang et al., 2007; Moore and Malinowski, 2009; Ainsworth et al., 2013), and possibly the interaction between orienting and conflict detection (Moore and Malinowski, 2009). However, only a few studies (Josefsson and Broberg, 2011; Ainsworth et al., 2013; Di Francesco et al., 2017; Jaiswal et al., 2018) have investigated the effects of dispositional mindfulness on the same attention networks. We applied the FFMQ that measures dispositional mindfulness as a multi-faceted construct and measured the attention networks with the ANT-R allowing for studying the interaction between early operating orienting and conflict detection. Thus, the FFMQ facets were expected to predict lower scores on the interaction of orienting by conflict detection. This expectation was confirmed, with dispositional mindfulness associating with a more efficient early operating ability to disengage from invalid cues to engage attention focus to detect the flanker conflict. This indicates that higher tendencies to be in a mindful state associates with more flexible attention orienting in everyday life. This flexible orienting makes it easier to disengage from salient stimuli/information that is irrelevant for goal-directed behavior. This positive effect of mindfulness on attention orienting was, similarly, reported in a recent meta-analysis of mindfulness induction in laboratory settings (Leyland et al., 2018). It was the FFMQ facets Describing and Non-Judgment that specifically predicted this flexibility of the orienting attention network, which facilitated an efficiency of the flanker incongruent conflict detection. Interestingly, the same two FFMQ facets were in a recent study shown to associate with neuroanatomical structures involved in attentional control and structures specifically involved in the interaction between orienting and conflict detection (Zhuang et al., 2017). Also, a previous study found the Describing facet to associate with higher attentional control abilities (Josefsson and Broberg, 2011). However, contrary to expectations, we did not find that the FFMQ facet of Acting with Awareness associated with attentional control.

The influential operational definition of mindfulness by Bishop et al. (2004) divides mindfulness into two components. One of the components is self-regulation of attention focus, such as the ability to sustain attention and to flexibly shift attention focus. Our results are in accordance with the operational definition that mindfulness associates with a more efficient ability to shift attention focus. Thus, it is highlighted that mindfulness is suggested to associate with higher abilities in cognitive inhibition, “particularly at the level of stimulus selection” (Bishop et al., 2004, p. 233). Bishop et al. (2004) referred to Posner’s paper from 1980 where he studied orienting attention, the attention network for stimulus selection, with a paradigm of valid and invalid cues. This paradigm is included in the revised version of the ANT (ANT-R), and not in the original ANT, and comprises the orienting network score in the current study (see Fan et al., 2009 for different approaches in assessing orienting attention). Further, it was previously shown that increased ability to sustain attention, as measured with a more efficient tonic alertness, is observed in experienced meditators but not following short-term mindful meditation training (Jha et al., 2007; Pagnoni and Cekic, 2007; MacLean et al., 2010; Tang et al., 2012). A systematic review of neuropsychological effects of mindfulness training (Chiesa et al., 2011) noted that early phases of mindfulness training seem to enhance conflict detection (executive control) and orienting, and later phases (longer time of meditation practice) alerting (see Tang et al., 2015). Therefore, we did not expect dispositional mindfulness to associate with higher tonic alertness (faster reaction times on the alertness network).

It has previously been highlighted (see Van Dam et al., 2011) that self-reports of dispositional mindfulness may be problematic because they tap into higher-order cognitive functions. It can thus be speculated that high scores on the Describing facet reflect high abilities to verbally describe and express (self-report) tendencies of being in mindful states. Interestingly, Zhuang et al. (2017) found the Describing facet to be the only FFMQ facet that correlated with brain areas that involve several of the mindfulness aspects of attentional control, self-awareness, and emotion regulation. Higher scores on the Describing facet may as such tap into higher meta-cognitive abilities following being high on dispositional mindfulness. Being in a mindful state is described as a meta-cognitive process by “both control of cognitive processes (i.e., attention self-regulation) and monitoring the stream of consciousness” (Bishop et al., 2004, p. 233).

Being attentive without judging is a core attitude of being mindful and may help an individual to preserve attentional resources for outer experiences. As such, it may increase the adaptive ability to flexibly adjust to situational contingencies. Our results seem to illustrate this relationship, with higher scores on the non-judgment facet associating with a higher flexibility of attention focus according to situational contingencies (i.e., lower scores on the orienting by conflict detection). For instance, in everyday life we all experience negative thoughts or emotions, or we feel tired. When we focus our energy on evaluating these reactions and relating to them as if they are somehow wrong or should not have been there, we will have less attention capacity for focusing on the task at hand at work or at school, or in conversation with others in social settings. The opposite of being non-judgmental may as such be that an individual focuses on interpreting and evaluating sensations and experiences, which may draw attentional resources from outer moment-to-moment experiences to these inner processes. This is for instance illustrated in several studies showing the reciprocal relationship between attention resources allocated for inner and outer experiences, of which intrinsic thought processing seem to lower the brain resources available for task performance (Weissman et al., 2006; Eichele et al., 2008; Sheline et al., 2009; Andrews-Hanna et al., 2014). Attention has a limited capacity (Schneider and Shiffrin, 1977) and our voluntarily ability to select attention focus in the presence of multiple stimuli, which most of us are surrounded by in our everyday life, seem to determine our ability to succeed in education, work, and in social interactions.

Interestingly, the FFMQ facets of Describing and Non-Judgment did not correlate with each other in the current study. So it seems that the Describing facet taps the meta-cognitive perspective of being mindful and Non-Judgment the attitudinal component of being mindful, both important for our self-regulation of attention (Bishop et al., 2004). Both of these facets correlated with the facet of Acting with Awareness, a facet we expected would predict higher attentional control abilities but did not show an association with attentional control abilities in the current study.

There is a general discussion of what mindfulness is, and how to define and operationalize it (see Van Dam et al., 2018). Following this discussion, and the general challenge of using instruments attempting to assess complex constructs, it has been noted limitations in how well questionnaires such as the FFMQ assesses all elements of the concept of mindfulness (see Grossman and Van Dam, 2011; Christopher et al., 2014; Williams et al., 2014). Therefore, our findings may not tap into all aspects of being mindful or be representative of effects on attentional control following mindfulness training.

## Conclusion

We are, to our best knowledge, the first to study the association between mindfulness and attentional control using the revised version of the ANT (ANT-R) developed by Fan et al. (2009). This version allows for the unique opportunity to study the interactions between the early operating attention allocation of orienting and conflict detection. Our results thus support that this task may provide new knowledge about the attentional mechanism of a tendency to be in mindful states. Being high on dispositional mindfulness associated with faster reactions on the interaction score of orienting by conflict detection. This indicates that being mindful allows for a more flexible and efficient orienting attention, enabling a quick process of selecting relevant stimuli in order to pursue goal-directed behavior (conflict detection).

## Data Availability

This dataset are available by contacting the corresponding author LS.

## Ethics Statement

The study was carried out in accordance with the recommendations and approval of the Regional Committee for Medical Research Ethics of South East Norway, University of Oslo, Norway, study number 2014/148. All subjects were informed about the study and gave a written informed consent in accordance with the Declaration of Helsinki.

## Author Contributions

LS, ES, BO, SA, and P-EB conceived and designed the study. LS, ES, BO, P-EB, JS and EV acquired the data. LS analyzed and interpreted the data, and wrote the manuscript. LS, BO, EV, JS, SA, P-EB, and ES critically reviewed the manuscript and agreed to be accountable for all aspects of the work.

## Conflict of Interest Statement

The authors declare that the research was conducted in the absence of any commercial or financial relationships that could be construed as a potential conflict of interest.

## Footnotes

1 We also conducted the multiple regression analyses with including the FFMQ and the ANT-R scores as standardized z-scores. These analyses produced the same results as including the FFMQ and the ANT-R scores as raw scores.
