# Supplementary material for: Dispositional Mindfulness and Attentional Control: The Specific Association Between the Mindfulness Facets of Non-judgment and Describing With Flexibility of Early Operating Orienting in Conflict Detection
Source: Front Psychol. 2018 Nov 29;9:2359. doi: 10.3389/fpsyg.2018.02359 (PMC6282922; doi:10.3389/fpsyg.2018.02359)
Supplement: Supplementary file 1 [file Table_1.DOCX]

Supplementary Table 1. Bivariate correlations between the ANT-R scores and the spatial cue condition trials (raw scores).

|  | Congruent Conflict Detection | | Incongruent Conflict Detection | |
| --- | --- | --- | --- | --- |
|  | Invalid cues | Valid cues | Invalid Cues | Valid cues |
| Alerting | -.16 | -.27 | -.07 | -.06 |
| Orienting | .38** | -.02 | .47** | -.06 |
| Conflict Detection | -.10 | -.03 | .52** | .66** |
| Alerting by Conflict Detection | -.07 | -.01 | -.01 | -.05 |
| Orienting by Conflict Detection | -.03 | -.05 | .41** | -.23 |
| Note. N = 50. ** = *p* < .01; * = *p* < .05. | | | | |
